# Heterotopic Ossification Circumferentia Articularis (HOCA) of Both Knee Joints After Guillain-Barré Syndrome

**DOI:** 10.7759/cureus.480

**Published:** 2016-02-05

**Authors:** Raju Vaishya, Amit Kumar Agarwal, Vipul Vijay, Abhishek Vaish

**Affiliations:** 1 Orthopaedics, Indraprastha Apollo Hospitals

**Keywords:** heterotopic ossification, myositis ossificans, knee, guillain-barré syndrome

## Abstract

Heterotopic ossification (HO) is the abnormal development of bone within soft tissue. It is a frequent complication after traumatic as well as atraumatic central nervous system (CNS) insult. It has rarely been found to be associated with Guillain-Barré syndrome (GBS). Only a few cases of HO associated with GBS have been reported so far in medical literature. We present a 30-year-old female patient with severe bilateral knee stiffness following axonal polyneuropathy type of GBS that developed 10 months ago in her immediate post-partum period. She was put on mechanical ventilation for two weeks. She was diagnosed as HO based on clinical and radiological studies. This is an extremely unusual presentation of HO encircling both the knees following GBS without any other well-known risk factors. We have coined a new nomenclature—Heterotopic Ossification Circumferentia Articularis (HOCA)—for this type of presentation. In our patient, various factors such as prolonged ICU stay, mechanical ventilation, hypoxia, and long-standing hypomobility could be attributed to the development of this severe form of HO.

## Introduction

Heterotopic ossification (HO) is an abnormal bone formation in the soft tissue. It is seen after traumatic as well as atraumatic neurologic injuries [1]. The incidence of HO ranges from 10%–25% [2]. The pathophysiology of HO is still not clear, but some risk factors that have been found to be associated with HO are: soft tissue trauma, neurologic injury, joint replacement surgery, tissue hypoxia, prolonged immobilization, prolonged bed rest, hypermetabolic status, and age over 60 years [3]. Guillain-Barré syndrome (GBS) is a type of autoimmune neuropathy, which is characterized by ascending progressive paralysis. The occurrence of HO in GBS has been sparsely reported in literature [4]. We report an extremely unusual presentation of HO encircling both the knees following GBS without any other well-known risk factors. This nomenclature has not been described in medical literature before. Informed patient consent was obtained for this study. 

## Case presentation

A 30-year-old female patient presented with severe bilateral knee stiffness, difficulty in walking, and mild pain in the left knee. She had been affected with a severe axonal polyneuropathy type of GBS in her immediate post-partum period after she delivered twin babies 10 months ago. She was on mechanical ventilation for two weeks and received intravenous immunoglobulins (IVIG), following which she recovered from this severe neurological illness over a period of three months. Gradually, however, she began to experience difficulty in walking and bending both knees.

**Figure 1:**
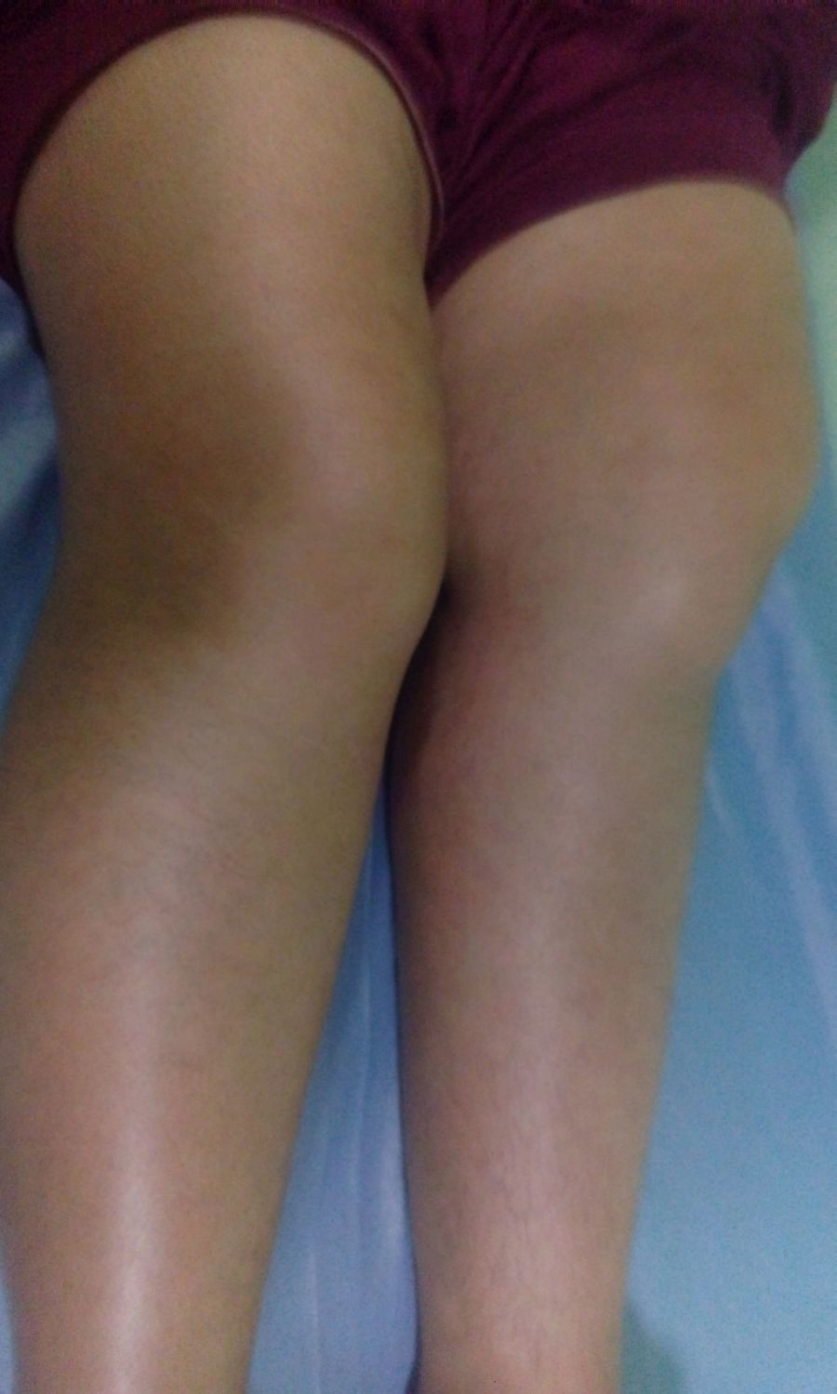
Clinical photograph of both lower limbs showing fixed flexion deformities

A physical examination revealed that both the knees had fixed flexion deformity of about 15 degrees (Figure 1), and there was only a jog of flexion in either knee.

**Figure 2:**
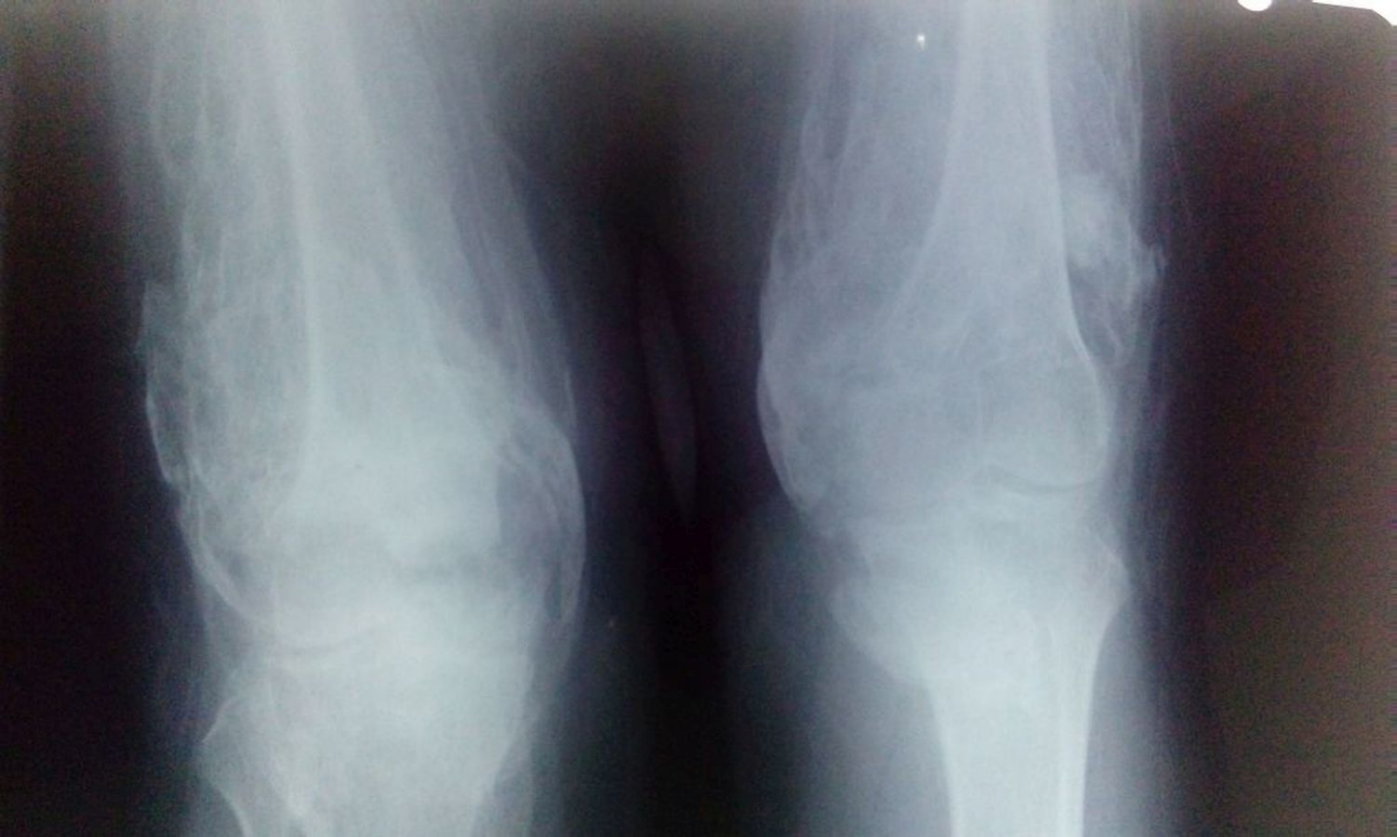
Anteroposterior radiograph of the knees showing diffuse heterotopic ossification

**Figure 3:**
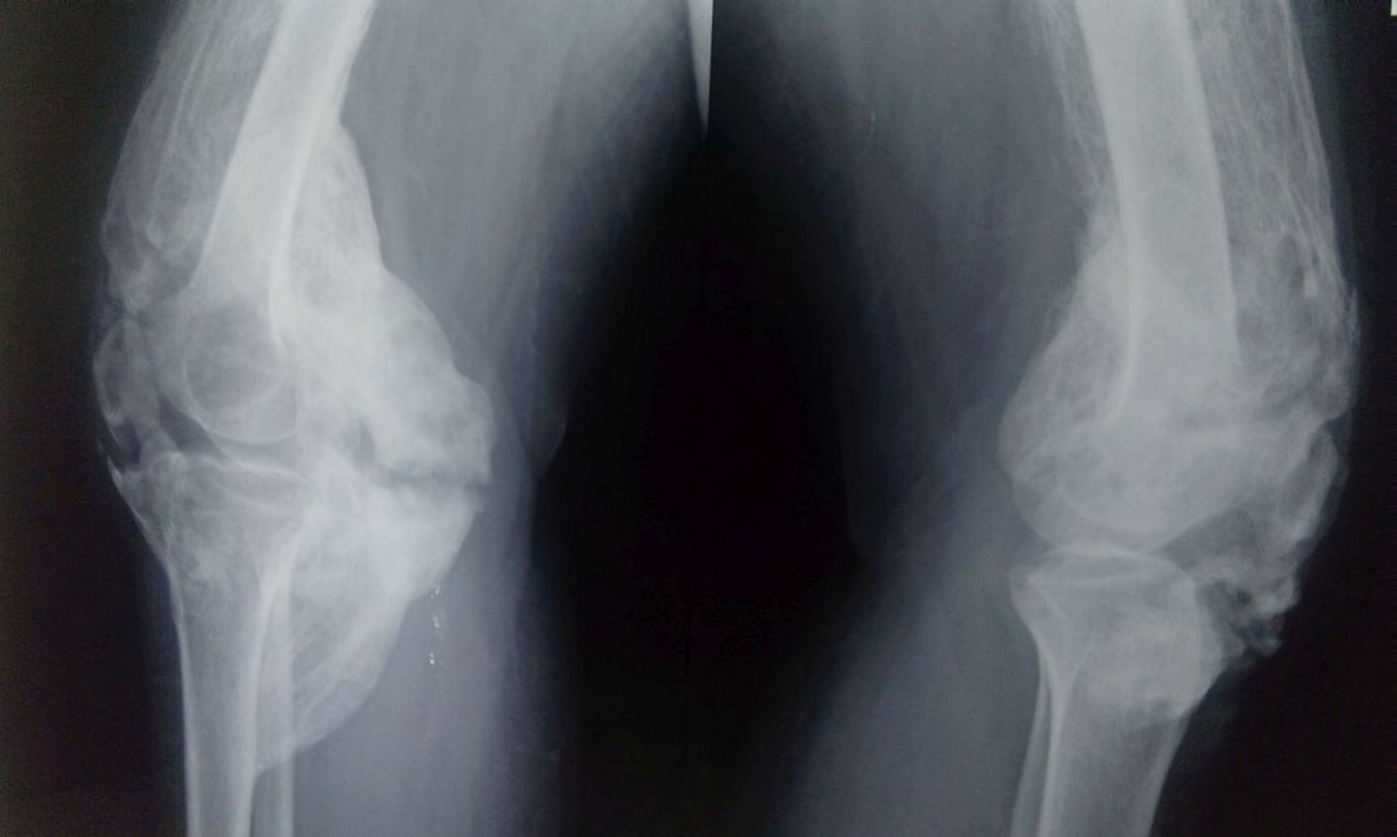
Lateral radiographs of the knees showing circumferential presence of diffuse heterotopic ossification

A hard bone mass was felt all around the knees with no involvement of the overlying skin. Plain radiographs revealed a diffuse heterotopic bone formation all around the knees (Figures 2-3).

A three-phase isotope bone scan using Technetium-99m-methylene diphosphonate (99mTC-MDP) showed an increased uptake around both the knees, suggestive of an inflammatory pathology with marked soft tissue involvement. She was diagnosed as HO based on the clinical findings and radiological studies. The patient was given intravenous zoledronic acid (5 mg/month) for six months and indomethacin (75 mg/day) for three months. Due to extensive involvement of the knees by HO, surgical excision was not contemplated. There was an improvement in the pain score, but the stiffness of the knees remained the same.

## Discussion

HO is a clinical condition characterised by abnormal mature bone formation in the soft tissue. This can occur after either trauma or after neurologic injury or disease, such as GBS. HO also tends to develop in trauma patients with the incidence ranging from 2%–7% [5].

The exact aetiopathogenesis of HO is still not known, and various hypotheses have been proposed. The underlying pathology of HO formation is inappropriate differentiation of fibroblasts into bone-forming cells. The dormant-lying osteoprogenitor stem cells in the soft tissues, under a proper stimulus, differentiate into osteoblasts that eventually lead to the formation of HO [6].

Several neurologic factors like coma, mechanical ventilation, and hypomobility with increased muscle tone could contribute to the formation of HO [7]. Although, HO has been found associated after GBS, no positive correlation between these two conditions has been found in the development of HO. We do not know whether HOCA is associated with only GBS or related to any other disease. Further studies are required to find the association of HOCA with any other disease.

GBS may present with pain, sensory symptoms, and autonomic involvement. The patient regains normal neurologic function in an estimated 80% of cases [8]. Only a few reports of HO occurring in GBS are available, although this complication is quite common after CNS trauma [9].  

The clinical presentation of HO in early stages includes pain and swelling of the affected area. A clinical diagnosis is often difficult in the initial phase and may require a three-phase bone scan to establish the diagnosis [10]. Serum alkaline phosphatase may also be elevated. However, in the advanced stage, diagnosis can be done by plain radiograph only. The differential diagnosis includes low grade osteosarcoma, deep vein thrombosis, joint inflammation, muscle tear with or without hematoma, fracture, etc. The larger joints like hip, elbow, and knee are usually affected by HO [11]. In severe cases, when it is present all around the joint it may be called as Heterotopic Ossification Circumferentia Articularis. This unique type of circumferential involvement of the joint has not been described in medical literature.

The treatment of HO is usually conservative in the initial phase of its development. Bisphosphonates have been found to be useful in the prevention as well as in the early stages. Bisphosphonates inhibit osteoid mineralization by binding calcium phosphate and by preventing hydroxyapatite crystallization [12]. Nonsteroidal anti-inflammatory drugs (NSAIDs) and radiation have been used as conservative treatment modalities in the management of HO. When HO significantly affects the range of motion (ROM) of the joint, surgical resection is recommended [13]. Surgical removal is rarely advocated, and only after the HO has fully matured, and this may take 12–18 months.

The incidence of HO in GBS patients appears to be common in ventilated patients with prolonged hospitalization. The development of HO seems to be due to not only a severe axonal injury requiring prolonged mechanical ventilation, but also the genetic predisposition of an individual, alterations in acid-base balance, and some unknown factors. There is a possibility that alterations of acid-base homeostasis during the mechanical ventilatory period may have a role to play in the pathophysiology by decreasing osteoclastic enzyme activity [14].

## Conclusions

Heterotopic Ossification Circumferentia Articularis (HOCA) is an extremely rare complication of late-onset HO after GBS in which there is circumferential involvement of the joint. We suggest that hypomobility with prolonged immobilization may be responsible for the development of late-onset HO after GBS. The aetiopathogenesis of HO is not clearly defined. It is necessary to recognize the symptoms and signs of HO early to prevent its progression.

## References

[1] Van Kuijk AA, Geurts AC, van Kuppevelt HJ (2002). Neurogenic heterotopic ossification in spinal cord injury. Spinal Cord.

[2] Garland DE (1991). A clinical perspective on common forms of acquired heterotopic ossification. Clin Orthop.

[3] Gardner MJ, Ong BC, Liporace F (2002). Orthopedic issues after cerebrovascular accident. Am J Orthop.

[4] Zeilig G, Ohry A, Shemesh Y (1988). The rehabilitation of patients with severe Guillain-Barre syndrome. Paraplegia.

[5] Jensen LL, Halar E, Little JW, Brooke MM (1987). Neurogenic heterotopic ossification1. Am J Phys Med.

[6] Jackson W, Aragon A, Djouad F (2009). Mesenchymal progenitor cells derived from traumatized human muscle. J Tissue Eng Regen Med.

[7] Citak M, Suero EM, Backhaus M (2012). Risk factors for heterotopic ossification in patients with spinal cord injury: a case–control study of 264 patients. Spine.

[8] Zeilig G, Weingarden HP, Levy R, Peer I, Ohry A, Blumen N (2006). Heterotopic ossification in Guillain-Barre syndrome: incidence and effects on the functional outcome with long-term follow-up. Arch Phys Med Rehabil.

[9] Kerdoncuff V, Sauleau P, Petrilli S, Duruflé A, Ben Beroukh K, Brissot R, Gallien P (2002). Heterotopic ossification in Guillain-Barré syndrome (article in French). Ann Phys Rehabil Med.

[10] Mielants H, Vanhove E, de Neels J, Veys E (1975). Clinical survey of and pathogenic approach to para-articular ossifications in long-term coma. Acta Orthop Scand.

[11] Wharton GW (1975). Heterotopic ossification. Clin Orthop Relat Res.

[12] Stover SL, Hataway CJ, Zeiger HE (1975). Heterotopic ossification in spinal cord-injured patients. Arch Phys Med Rehabil.

[13] Pape HC, Marsh S, Krettek C (2004). Current concepts in the development of heterotopic ossification. J Bone Joint Surg Br.

[14] Bushinsky DA (1996). Metabolic alkalosis decreases bone calcium efflux by suppressing osteoclasts and stimulating osteoblasts. Am J Physiol.

